# The Clinical Value of Poor-Law Infirmaries

**Published:** 1915-02-13

**Authors:** 


*To the Editor of* The Hospital.

### The Clinical Value of Poor-Law Infirmaries.

*To the Editor of* The Hospital.

Sir,—The importance of this subject is fully demonstrated by the readiness with which the "rejoinder" followed. The real clinical value of the material in Poor-Law infirmaries has not yet been sufficiently realised. The object of my short article was to show the too little attention now given to these valuable opportunities and to plead for a more general and systematic recording of notes. In spite of all that has been written in reply, I adhere to the great bulk of what I have written and present it to your readers as a true record of my Poor-Law experience, direct and indirect.

There are, of course, Poor-Law infirmaries and Poor-Law infirmaries; there are also criticisms and criticisms. The reply of Dr. Stewart (in spite of an occasional tendency to a personal gibe) is a most reasonable one and quite a difficult criticism to meet. I regret that I cannot say as much for the "rejoinder of a Medical Superintendent," which I propose to take first.

His reply is really one of special pleading. He reiterates some of my main points, such as the "paucity of the staff" and the difference between the records of infirmaries and the voluntary hospitals. So far we are evidently in agreement. But he evidently considers my conclusions unjust. His criticism, however, borders on the side of puerility and "seems" only for the purpose of the destructive criticism of the article. For example, I wrote "Paid doctors are being required by the Insurance Act to do a considerable amount of clerical work and to take notes and records of their diagnoses and treatment and number of visits as data for possible future statistics." A "Medical Superintendent" writes: "This is not the case." And then he proceeds, "The only records the panel doctor is required to keep are a bare statement of the name, sex, age, and occupation of the patient, the diagnosis, and the dates of his attendances." Is this not considerable clerical work? "No notes of the physical examination, of the progress of the case, or of the treatment are required from him." But diagnoses of the cases are made and recorded by a busy practitioner, and a record of his treatment can usually be obtained from his prescription books. Besides, that is not the point. My obvious point was that much more was expected from institutions than from private practitioners, and when even panel doctors have to take such notes as they do take it is incumbent on Poor-Law institutions to look into this matter of note-recording more thoroughly. I think the gist of this meaning can be seen throughout the article.

His treatment of the details of the carbonic-acid poisoning incident is amusing and very illustrative. He pleads that the sum total of notes on the card—"admitted as a supposed case of gas-poisoning—*seems* well enough now"—is sufficient. When a medical superintendent exists who defends publicly such laxity in records, one begins to realise the real need, as well as the difficulties, of Poor-Law medical administration reform. I submit that in this case such a record was more than useless. The word "seems" does imply only judgment from external appearance. A thorough examination of the poor woman should have been made, with definite notes on every system, especially on the blood and nervous system. I hold to that in spite of every sneer about pleasure to the official formalist or the fashion of hitting infirmaries. The pallor, emaciation, debility, and nervousness of this destitute woman in court were striking. The "as far as I recollect" basis is not good enough evidence in such cases as a rule. Dilating on medical honour or dishonour does not help the special-pleading scribe. Either notes should have been made or they should not.

The name, age, sex, occupation, and religion and other particulars required by regulation are usually done by the clerical staff, and the case-sheet with such details filled in meets the R.M.O. in the receiving room. It is sufficiently obvious that my article refers to the clinical notes, such as previous history and condition on admission, and these are the charge of the medical officer who receives or who treats the case.

A "Medical Superintendent's" reply on the autopsy register is on similar lines. I plead for a "book specially kept for this purpose and signed by the officer performing the autopsy," and my remarks are "incorrect," as "records are made on the patient's case-sheet" or a "copy of the official form" is so attached. I feel I can safely leave my article to be read against his rejoinder.

In quite another street is the reply of Dr. Stewart. I freely admit that from his description the system in vogue in Paddington Infirmary (even excluding the wealth of administrative, as apart from clinical, details of the system supplied) seems admirable. Fancy, such an excellent scheme has actually been in practice for thirty years! Has it been adopted in all infirmaries, and if not, why not?

Human nature has a tendency to regard all general criticism as particular and personal. It must not be forgotten that many institutions of this kind exist, and criticisms which may be strong to the worst are only just to the average, and tend to be unfair to the best-managed institutions. But one must strike an average. Mere length of annual report means nothing. In the M.A.B. reports many of the superintendents' observations are, for many reasons, according to circumstances, omitted. And the statistical part of the work must be considered, for statistics are supplied from each institution to the statistical committee for rearrangement and completion. Besides, this was a mere unemphasised side-issue to my article. The M.A.B. medical reports are issued in one volume and published. Poor-Law reports one would have to obtain, not from a central source in one volume, but from different Poor-Law districts.

The bias of the poor against the infirmary still exists, and is only gradually breaking down. Local considerations modify it, sometimes to a considerable extent; as knowledge spreads and improvements and reforms continue it will disappear.

I was fortunate enough to see the syllabus which Dr. Stewart prepared last year for the (London) Medical Society, and was asked by a member to accompany him to the meeting. The syllabus was an excellent one, and I understand the results exhibited were very instructive and interesting. Such meetings are invaluable for the infirmary, and for the inauguration of such meetings (I had never heard of a similar one in an infirmary) Dr. Stewart deserves the congratulation of every Poor-Law enthusiast. Of course, "every courtesy" is shown to the infirmary staff, I hope, on every occasion. As I said before, it is not the staff but the system that is at fault. As an infirmary officer I was overworked, nearly run off my feet, and could never find time to do the work as thoroughly and as conscientiously as I would have liked. The contempt is not directed against any particular infirmary or staff; but even if it is called by a more euphemistic name, still it exists. A young infirmary officer soon finds this out during his canvass for one of the posts in the voluntary hospitals.—Yours faithfully,

The Writer of the Article.

February 8, 1915.

